# Supplementary material for: A JNK-interacting protein 1 acts across the midline to mediate synaptic localization of the SARM1 calcium-signaling scaffold protein for asymmetric neuronal fate choice
Source: bioRxiv. 2026 May 5:2026.04.30.722091. Preprint. [Version 1] doi: 10.64898/2026.04.30.722091 (PMC13174285; doi:10.64898/2026.04.30.722091)
Supplement: 1 [file NIHPP2026.04.30.722091v1-supplement-1.pdf]

bioRxiv preprint doi: https://doi.org/10.64898/2026.04.30.722091; this version posted May 5, 2026. The copyright holder for this preprint (which was not certified by peer review) is the author/funder, who has granted bioRxiv a license to display the preprint in perpetuity. It is made available under aCC-BY 4.0 International license.

Supplemental Information Appendix

Supplemental Materials and Methods

Supplemental References

Supplemental Figure Legends

Supplemental Figures

bioRxiv preprint doi: https://doi.org/10.64898/2026.04.30.722091; this version posted May 5, 2026. The copyright holder for this preprint (which was not certified by peer review) is the author/funder, who has granted bioRxiv a license to display the preprint in perpetuity. It is made available under aCC-BY 4.0 International license.

Supplemental Materials and Methods

Strains and transgenes

Animal protocols approved by the Office of Animal Care and Institutional Biosafety Committees at the University of Illinois Chicago were followed. Hermaphrodites of *C. elegans* were analyzed and imaged.

<u>*Mutants*</u>

nsy-5(ky634) I (CHUANG *et al.* 2007)

jip-1(vy6) II (This study)

jip-1(gk133506) II (THOMPSON *et al.* 2013)

jip-1(gk466982) II (THOMPSON *et al.* 2013)

ttTi5605 II (FROKJAER-JENSEN *et al.* 2008; FROKJAER-JENSEN *et al.* 2012)

unc-104(e1265) II (KUMAR *et al.* 2010)

tir-1(ky388ts) III (CHUANG AND BARGMANN 2005)

unc-36(e251) III (BRENNER 1974)

unc-116(e2310) III (PATEL *et al.* 1993)

unc-119(ed3) III (FROKJAER-JENSEN *et al.* 2008; FROKJAER-JENSEN *et al.* 2012)

nsy-4(ky627) IV (VANHOVEN *et al.* 2006)

unc-43(n1186lf) IV (PARK AND HORVITZ 1986)

cxTi10816 IV (FROKJAER-JENSEN *et al.* 2008; FROKJAER-JENSEN *et al.* 2012)

<u>*mNG knock-in strains*</u>

jip-1(vy260 [mNG::SEC::jip-1a/d knock-in]) II (this study)

bioRxiv preprint doi: https://doi.org/10.64898/2026.04.30.722091; this version posted May 5, 2026. The copyright holder for this preprint (which was not certified by peer review) is the author/funder, who has granted bioRxiv a license to display the preprint in perpetuity. It is made available under aCC-BY 4.0 International license.

jip-1(vy260 vy263 [mNG::jip-1a/d knock-in]) II (this study)

jip-1(vy286 vy290 [mNG::jip-1a/d(vy6) knock-in]) II (this study)

mNG knock-out strains

jip-1(vy308 [jip-1a/dE1-19 knock-out]) II (this study)

jip-1(vy309 [jip-1a/dE12-19 knock-out]) II (this study)

Integrated transgenes

Integrated transgenes	Figures
vyIs62 [*odr-3p::tir-1::GFP; ofm-1p::DsRed*] X (SIETE *et al.* 2024)	1, 4, S1
vySi50 [*odr-3p::tir-1::TagRFP; unc-119(+)*] IV (this study)	1
kyIs140 [*str-2p::GFP; lin-15(+)*] I (TROEMEL *et al.* 1999)	1, 3
vySi88 [*odr-3p::GFP::jip-1a; unc-119(+)*] II (this study)	1, 2
vyIs56 [*odr-1p::TagRFP*] III (COCHELLA *et al.* 2014)	2, 3
vySi97 [*odr-3p::GFP::jip-1a(vy6); unc-119(+)*] II (this study)	2
vyIs96 [*str-2p::TagRFP*] V (XIONG *et al.* 2026)	3
vySi55 [*ceh-36p::myrTagRFP; unc-119(+)*] IV (ALQADAH *et al.* 2019)	3

Extrachromosomal arrays

Extrachromosomal arrays	Figures
vyEx2266, 2267*, 2268** [*jip-1p::jip-1a* (3 ng/µl); *odr-1p::DsRed* (15 ng/µl); *ofm-1p::DsRed* (30 ng/µl)]	1
vyEx2271, 2272*, 2297, 2298*, 2299** [*odr-3p::jip-1a* (3 ng/µl); *odr-1p::DsRed* (15 ng/µl); *ofm-1p::DsRed* (30 ng/µl)]	1
vyEx2273, 2274** [*odr-3p::GFP::jip-1a* (3 ng/µl); *odr-1p::DsRed* (15 ng/µl); *ofm-1p::DsRed* (30 ng/µl)]	1

bioRxiv preprint doi: https://doi.org/10.64898/2026.04.30.722091; this version posted May 5, 2026. The copyright holder for this preprint (which was not certified by peer review) is the author/funder, who has granted bioRxiv a license to display the preprint in perpetuity. It is made available under aCC-BY 4.0 International license.

vyEx2294, 2295*, 2296** [*odr-3p::jip-1d* (3 ng/µl); *odr-1p::DsRed* (15 ng/µl); *ofm-1p::DsRed* (30 ng/µl)]	1
vyEx2275, 2276*, 2277*, 2278*, 2279*, 2280*, 2281** [*odr-3p::jip-1e* (3 ng/µl); *odr-1p::DsRed* (15 ng/µl); *ofm-1p::DsRed* (30 ng/µl)]	1
vyEx2282, 2283*, 2284*, 2285*, 2286*, 2287** [*odr-3p::jip-1f* (3 ng/µl); *odr-1p::DsRed* (15 ng/µl); *ofm-1p::DsRed* (30 ng/µl)]	1
vyEx2288, 2289*, 2290*, 2291*, 2292*, 2293** [*odr-3p::mJIP-1* (3 ng/µl); *odr-1p::DsRed* (15 ng/µl); *ofm-1p::DsRed* (30 ng/µl)]	1
vyEx1442 [*jip-1p::GFP* (20 ng/µl); *ofm-1p::DsRed* (30 ng/µl)]	3
vyEx1782, 1804 [*odr-3p::jip-1a* (3 ng/µl); *odr-1p::DsRed* (15 ng/µl); *ofm-1p::DsRed* (30 ng/µl)]	3
vyEx2472 [*odr-3p::GFP::jip-1a* (18 ng/µl); *odr-3p::unc-104::TagRFP* (15 ng/µl); *ofm-1p::DsRed* (30 ng/µl)]	5
vyEx2759 [*odr-3p::TagRFP::jip-1a* (18 ng/µl); *odr-3p::unc-116::GFP* (1 ng/µl); *ofm-1p::DsRed* (30 ng/µl)]	5
vyEx2749 [*odr-3p::GFP::jip-1a* (18 ng/µl); *odr-3p::tir-1::TagRFP* (15 ng/µl); *ofm-1p::DsRed* (30 ng/µl)]	5
vyEx611 [*odr-3p::GFP* (7.5 ng/µl); *ofm-1p::DsRed* (30 ng/µl)] (HSIEH *et al.* 2012)	S1
vyEx2720 [*odr-3p::GFP::jip-1a* (18 ng/µl); *ofm-1p::DsRed* (30 ng/µl)]	S3
vyEx2738 [*odr-3p::GFP::jip-1a(vy6)* (18 ng/µl); *ofm-1p::DsRed* (30 ng/µl)]	S3

*The *tir-1(ky388ts) and jip-1(vy6)* strains containing these extrachromosomal arrays did not propagate well and became sterile after a couple of generations. Thus, these lines were not frozen.

bioRxiv preprint doi: https://doi.org/10.64898/2026.04.30.722091; this version posted May 5, 2026. The copyright holder for this preprint (which was not certified by peer review) is the author/funder, who has granted bioRxiv a license to display the preprint in perpetuity. It is made available under aCC-BY 4.0 International license.

Plasmid construction

pCFJ356::odr-3p::tir-1::TagRFP was made by subcloning 7198 bp of *odr-3p::tir-1::TagRFP* into the pCFJ356 vector (FROKJAER-JENSEN *et al.* 2012).

jip-1p::jip-1a was generated by subcloning 3003 bp of *jip-1a* cDNA into a vector containing 3298 bp of the *jip-1* promoter.

odr-3p::jip-1a was generated by subcloning 3003 bp of *jip-1a* cDNA into a vector containing the *odr-3* promoter.

odr-3p::jip-1d was generated by deleting the 15 bp sequence (ccttcttctctccag) specific for *jip-1a/e/f* isoforms from *odr-3p::jip-1a* using Q5 site-directed mutagenesis kit (New England Biolabs)

odr-3p::jip-1e was generated by subcloning 1581 bp of *jip-1e* cDNA into a vector containing the *odr-3* promoter using Gibson assembly (New England Biolabs).

odr-3p::jip-1f was generated by subcloning 1119 bp of *jip-1f* cDNA into a vector containing the *odr-3* promoter using Gibson assembly (New England Biolabs).

odr-3p::GFP::jip-1a was generated by subcloning GFP into the 5' end of *jip-1a* cDNA in the *odr-3*p*::jip-1a* vector using Gibson assembly (New England Biolabs).

bioRxiv preprint doi: https://doi.org/10.64898/2026.04.30.722091; this version posted May 5, 2026. The copyright holder for this preprint (which was not certified by peer review) is the author/funder, who has granted bioRxiv a license to display the preprint in perpetuity. It is made available under aCC-BY 4.0 International license.

odr-3p::GFP::jip-1a(vy6) was generated by subcloning GFP into the 5' end of *jip-1a(vy6)* in the *odr-3*p*::jip-1a(vy6*) vector using Gibson assembly (New England Biolabs).

odr-3p::mJIP-1 was generated by subcloning 2094 bp of mJIP-1(MAPK8IP1) cDNA into a vector containing the *odr-3* promoter.

pAB1.1::*odr-3p::GFP::jip-1a* was made by subcloning 7414 bp of *odr-3p::GFP::jip-1a* into the *pAB1.1* vector (ALQADAH *et al.* 2016).

pAB1.1::*odr-3p::GFP::jip-1a(vy6)* was made by subcloning 7414 bp of *odr-3p::GFP::jip-1a(vy6)* was subcloned into the *pAB1.1* vector (ALQADAH *et al.* 2016).

odr-3p::TagRFP::jip-1a was generated by subcloning 1643 bp of *TagRFP* into a vector containing the *odr-3p* promoter and *jip-1a* cDNA.

sgRNA constructs for Cas9-triggered homologous recombination were made by subcloning the sgRNA fragment into the *pDD162 eft-3p::Cas9::empty sgRNA* vector as previously described (DICKINSON *et al.* 2013; DICKINSON *et al.* 2015; DICKINSON AND GOLDSTEIN 2016) (Addgene #47549 from Bob Goldstein's lab) using the Q5 site-directed mutagenesis kit (New England Biolabs).

Homology repair template constructs for Cas9-triggered homologous recombination were generated by subcloning 5' and 3' homology regions, generated by PCR, into the

bioRxiv preprint doi: https://doi.org/10.64898/2026.04.30.722091; this version posted May 5, 2026. The copyright holder for this preprint (which was not certified by peer review) is the author/funder, who has granted bioRxiv a license to display the preprint in perpetuity. It is made available under aCC-BY 4.0 International license.

pDD268 mNG::SEC::3xFLAG vector (DICKINSON *et al.* 2013; DICKINSON *et al.* 2015; DICKINSON AND GOLDSTEIN 2016) using Gibson Assembly (New England Biolabs). One or both guanine nucleotides of the PAM (protospacer adjacent motif, NGG motif) site, if present in the homology fragment of the repair template construct, were mutated using a Q5 site-directed mutagenesis kit (New England Biolabs).

The sequence of the gRNA fragment cloned into *pDD162 eft-3p::Cas9::empty sgRNA* vector, primers used to PCR amplify homology regions, and the location of the mutated nucleotide(s) of the PAM site are listed below for individual knock-in and knock-out strains.

mNG::SEC::jip-1a/d knock-in

eft-3p::Cas9::U6p::jip-1p sgRNA2 (GCCGACGGTTATTCGGAGGGT)

1759 bp of 5' homology region

Forward primer: GTGAAATGAGGCGCTGAACTTG

Reverse primer: CGATTTGTGCTGAAATTTTTAAGACTTAAAATTTCAAG

1558 bp of 3' homology region

Forward primer: ATGAGCGCATTTGAATGTCGAAAATG

Reverse primer: CTGGCAAATATTGGTCTGACGGACTAC

bioRxiv preprint doi: https://doi.org/10.64898/2026.04.30.722091; this version posted May 5, 2026. The copyright holder for this preprint (which was not certified by peer review) is the author/funder, who has granted bioRxiv a license to display the preprint in perpetuity. It is made available under aCC-BY 4.0 International license.

The GG nucleotides of the PAM site, located at 100 bp and 101 bp downstream of the *jip-1a/d* start codon within the 3' homology region, were mutated to CC to generate a silent mutation.

mNG::SEC::jip-1a/d (vy6) knock-in repair template construct was generated by mutagenizing *mNG::SEC::jip-1a/d knock-in* repair template construct using Q5 site-directed mutagenesis kit (New England Biolabs) with forward primer (AATTTCTGGTTCATCCGTGTCTTCG) and reverse primer (TGGCGTCAGAATCCTCTTCTGAATC).

jip-1a/dE1-19 knock-out

eft-3p::Cas9::U6p::jip-1p sgRNA2 (GCCGACGGTTATTCGGAGGGT)

eft-3p::Cas9::U6p::jip-1 sgRNA (GTAACCCAGAGAGGAGATCT)

1749 bp of 5' homology region

Forward primer: GTGAAATGAGGCGCTGAACTTG

Reverse primer: CGATTTGTGCTGAAATTTTTAAGACTTAAAATTTCAAG

1515 bp of 3' homology region

Forward primer: TAATTTTTTTTTAATTTTAAAATCAATTTGTCATCC

Reverse primer: CTCCAAGAGTACGCAAACATCTCAC

jip-1a/dE12-19 knock-out

bioRxiv preprint doi: https://doi.org/10.64898/2026.04.30.722091; this version posted May 5, 2026. The copyright holder for this preprint (which was not certified by peer review) is the author/funder, who has granted bioRxiv a license to display the preprint in perpetuity. It is made available under aCC-BY 4.0 International license.

eft-3p::Cas9::U6p::jip-1 E12-19 sgRNA (GCATCGGAATACGGCGGAGCA)

eft-3p::Cas9::U6p::jip-1 sgRNA (GTAACCCAGAGAGGAGATCT)

1709 bp of 5' homology region

Forward primer: TCTCAGCTGTAACAACAGTTTTGTGTAC

Reverse primer: GGATGGGAGTACCGATTGGAATAC

1515 bp of 3' homology region

Forward primer: TAATTTTTTTTTAATTTTAAAATCAATTTGTCATCC

Reverse primer: CTCCAAGAGTACGCAAACATCTCAC

Germline transformation

DNA was injected into the syncytial gonad of adult hermaphrodites (P_0) as described previously (MELLO AND FIRE 1995). F_1 progenies expressing the injected fluorescent transgenes were identified and cloned (1 animal per plate), and the F_2 progenies were screened for transgenic lines.

Genetic mosaic analysis

Genetic mosaic analysis was performed in animals containing unstable extrachromosomal transgenic arrays that had been passed for at least 6 generations before analysis, as described previously (SAGASTI *et al.* 2001; VANHOVEN *et al.* 2006). The mosaic animals that lose the extrachromosomal transgene in one of the two AWC cells

bioRxiv preprint doi: https://doi.org/10.64898/2026.04.30.722091; this version posted May 5, 2026. The copyright holder for this preprint (which was not certified by peer review) is the author/funder, who has granted bioRxiv a license to display the preprint in perpetuity. It is made available under aCC-BY 4.0 International license.

were identified by the loss of the co-injection marker *odr-1p::DsRed* (expressed in both AWC neurons in non-mosaic animals) expression in AWC.

Mos1-mediated single copy insertion (MosSCI)

Mos1-mediated single-copy insertion of transgenes was performed as described previously (FROKJAER-JENSEN *et al.* 2008). *pAB1.1*::*odr-3p::GFP::jip-1a* (33 ng/µl) or *pAB1.1*::*odr-3p::GFP::jip-1a(vy6)* (45 ng/µl) was injected into *ttTi5605* II; *unc-119(ed3)* III animals. *pCFJ356::odr-3p::tir-1::TagRFP* (67 ng/µl) was injected into *cxTi10816* IV*; unc-119(ed3)* III animals. Each of these transgenes was co-injected with *eft-3p::mos-1* (50 ng/µl), *hsp16.4p::peel-1* (10 ng/µl), *rab-3p::mCherry* (10 ng/µl), *myo-3p::mCherry* (5 ng/µl), and *myo-2p::mCherry* (2.5 ng/µl). The injected animals were grown at 25°C until the worms on the plates were starved. Animals were then heat shocked at 34°C for two hours to induce the expression of the negative selection marker PEEL-1, which kills animals carrying non-integrated transgenes. Animals that moved freely, resulted from the rescue of the *unc-119(ed3)* phenotype, and lost the expression of co-injection mCherry markers were cloned on single plates and verified by PCR to establish single-copy insertion lines.

Generation of mNG knock-in and knock-out strains using Cas9-triggered homologous recombination

mNG knock-in or knock-out strains were generated using Cas9-triggered homologous recombination as previously described (DICKINSON *et al.* 2013; DICKINSON *et al.* 2015; DICKINSON AND GOLDSTEIN 2016). The sgRNA/Cas9-expressing construct (50 ng/µl) and

bioRxiv preprint doi: https://doi.org/10.64898/2026.04.30.722091; this version posted May 5, 2026. The copyright holder for this preprint (which was not certified by peer review) is the author/funder, who has granted bioRxiv a license to display the preprint in perpetuity. It is made available under aCC-BY 4.0 International license.

homology repair template construct (50 ng/µl) were co-injected with three markers, *rab-3p::mCherry* (10 ng/µl), *myo-2p::mCherry* (2.5 ng/µl), and *myo-3p::mCherry* (5 ng/µl), into N2 animals cultured at 20 °C. The repair template construct's SEC cassette, flanked by LoxP sites, contains transcriptional terminators, a dominant-roller phenotype marker *sqt-1(e1350),* Cre driven by a heat-shock promoter, and a hygromycin resistance gene. Injected worms were allowed to lay eggs at 25 °C. Hygromycin (Gold Biotechnology) was applied directly to the plates at a final concentration of 250 µg/ml three days later, and the plates were cultured at 25 °C. The animals that showed a roller phenotype and lost red fluorescent co-injection markers were cloned onto single plates three to four days later to establish insertion lines. L1 progenies of the insertion lines were heat shocked at 34°C for four hours to induce Cre expression, then cultured at 20 °C for 5-7 days. The F_2 progeny of heat-shocked worms that moved freely (non-rollers) and expressed mNG were cloned to establish SEC-excised lines. The correct insertion of the mNG marker and the correct excision of SEC were verified by PCR and sequencing.

Live imaging of transgenic animals expressing fluorescent proteins

Animals, anesthetized with 5 mM sodium azide (Sigma) or 7.5 mM levamisole (Sigma), were mounted on 2% agarose pads on microscope slides. Images were obtained using a Zeiss Axio Imager M2 microscope, equipped with a motorized focus drive, a Zeiss objective EC Plan-Neofluar 40x/1.30 Oil DIC M27, a Piston GFP bandpass filter set (41025, Chroma Technology), a TRITC filter set (41002c, Chroma Technology), a Zeiss AxioCam 506 mono CCD digital camera or a Hamamatsu digital camera C11440, and a Zeiss Apotome system. Images were captured using Zeiss ZEN imaging software.

bioRxiv preprint doi: https://doi.org/10.64898/2026.04.30.722091; this version posted May 5, 2026. The copyright holder for this preprint (which was not certified by peer review) is the author/funder, who has granted bioRxiv a license to display the preprint in perpetuity. It is made available under aCC-BY 4.0 International license.

Quantification of fluorescence intensity

Animals from each set of experiments were imaged at L1 with the same exposure time. Fluorescence intensity was measured using ImageJ or Zeiss ZEN. In Figures 2D, S1A, and S1B, GFP fluorescence intensity in the AWC axon and cell body was measured in the maximum intensity projection of Z-stack images. In Figures 3B, 3D, 3F, and S1D, a single focal plane with the brightest fluorescence in the AWC cell body was selected from a stack of images to compare fluorescence intensity. mNG and GFP fluorescence intensity were normalized by the TagRFP intensity measured in the same AWC cell in Figures 3B and 3D, respectively.

Time-lapse imaging of protein trafficking

Worms in the L1 larval stage were anesthetized with 7.5 mM tetramisole (Sigma) and mounted onto 2% agarose pads on microscope slides for imaging. Our previous study showed that 7.5 mM tetramisole, compared to 0.5 mM, 1 mM, and 2 mM, did not significantly affect the axonal transport of TIR-1::GFP along AWC axons (SIETE *et al.* 2024). Time-lapse images were acquired for 30 seconds with a speed of 5 (TIR-1::GFP) or 10 (GFP::JIP-1a and GFP::JIP-1a^{E58K}) frames per second and an exposure time of 100 (GFP::JIP-1a and GFP::JIP-1a^{E58K}) or 200 (TIR-1::GFP) milliseconds using a Zeiss Axio Imager M2 microscope equipped with a Zeiss objective EC Plan-Neofluar 40x/1.30 Oil DIC M27, EC Plan-Neofluar 63x/1.40 Oil DIC M27, a Piston GFP bandpass filter set (41025, Chroma Technology), and a Hamamatsu digital camera C11440. Acquired images were analyzed to generate kymographs using Fiji (SCHINDELIN *et al.* 2012) with

bioRxiv preprint doi: https://doi.org/10.64898/2026.04.30.722091; this version posted May 5, 2026. The copyright holder for this preprint (which was not certified by peer review) is the author/funder, who has granted bioRxiv a license to display the preprint in perpetuity. It is made available under aCC-BY 4.0 International license.

the KymographClear macro (version 2.0a) (MANGEOL *et al.* 2016). The percentage and velocity of moving events were measured using KymographDirect (version 2.1) (MANGEOL *et al.* 2016).

Supplemental references

Alqadah, A., Y. W. Hsieh, J. A. Schumacher, X. Wang, S. A. Merrill *et al.*, 2016 SLO BK Potassium Channels Couple Gap Junctions to Inhibition of Calcium Signaling in Olfactory Neuron Diversification. PLoS Genet 12**:** e1005654.

Alqadah, A., Y. W. Hsieh, R. Xiong, B. J. Lesch, C. Chang *et al.*, 2019 A universal transportin protein drives stochastic choice of olfactory neurons via specific nuclear import of a sox-2-activating factor. Proc Natl Acad Sci U S A 116**:** 25137–25146.

Brenner, S., 1974 The genetics of Caenorhabditis elegans. Genetics 77**:** 71–94.

Chuang, C. F., and C. I. Bargmann, 2005 A Toll-interleukin 1 repeat protein at the synapse specifies asymmetric odorant receptor expression via ASK1 MAPKKK signaling. Genes Dev 19**:** 270–281.

Chuang, C. F., M. K. Vanhoven, R. D. Fetter, V. K. Verselis and C. I. Bargmann, 2007 An innexin-dependent cell network establishes left-right neuronal asymmetry in C. elegans. Cell 129**:** 787–799.

Cochella, L., B. Tursun, Y. W. Hsieh, S. Galindo, R. J. Johnston *et al.*, 2014 Two distinct types of neuronal asymmetries are controlled by the Caenorhabditis elegans zinc finger transcription factor die-1. Genes Dev 28**:** 34–43.

bioRxiv preprint doi: https://doi.org/10.64898/2026.04.30.722091; this version posted May 5, 2026. The copyright holder for this preprint (which was not certified by peer review) is the author/funder, who has granted bioRxiv a license to display the preprint in perpetuity. It is made available under aCC-BY 4.0 International license.

Dickinson, D. J., and B. Goldstein, 2016 CRISPR-Based Methods for Caenorhabditis elegans Genome Engineering. Genetics 202**:** 885–901.

Dickinson, D. J., A. M. Pani, J. K. Heppert, C. D. Higgins and B. Goldstein, 2015 Streamlined Genome Engineering with a Self-Excising Drug Selection Cassette. Genetics 200**:** 1035–1049.

Dickinson, D. J., J. D. Ward, D. J. Reiner and B. Goldstein, 2013 Engineering the Caenorhabditis elegans genome using Cas9-triggered homologous recombination. Nat Methods 10**:** 1028–1034.

Frokjaer-Jensen, C., M. W. Davis, M. Ailion and E. M. Jorgensen, 2012 Improved Mos1-mediated transgenesis in C. elegans. Nat Methods 9**:** 117–118.

Frokjaer-Jensen, C., M. W. Davis, C. E. Hopkins, B. J. Newman, J. M. Thummel *et al.*, 2008 Single-copy insertion of transgenes in Caenorhabditis elegans. Nat Genet 40**:** 1375–1383.

Hsieh, Y. W., C. Chang and C. F. Chuang, 2012 The microRNA mir-71 inhibits calcium signaling by targeting the TIR-1/Sarm1 adaptor protein to control stochastic L/R neuronal asymmetry in C. elegans. PLoS Genet 8**:** e1002864.

Kumar, J., B. C. Choudhary, R. Metpally, Q. Zheng, M. L. Nonet *et al.*, 2010 The Caenorhabditis elegans Kinesin-3 motor UNC-104/KIF1A is degraded upon loss of specific binding to cargo. PLoS Genet 6**:** e1001200.

Mangeol, P., B. Prevo and E. J. Peterman, 2016 KymographClear and KymographDirect: two tools for the automated quantitative analysis of molecular and cellular dynamics using kymographs. Mol Biol Cell 27**:** 1948–1957.

Mello, C., and A. Fire, 1995 DNA transformation. Methods Cell Biol 48**:** 451–482.

bioRxiv preprint doi: https://doi.org/10.64898/2026.04.30.722091; this version posted May 5, 2026. The copyright holder for this preprint (which was not certified by peer review) is the author/funder, who has granted bioRxiv a license to display the preprint in perpetuity. It is made available under aCC-BY 4.0 International license.

Park, E. C., and H. R. Horvitz, 1986 Mutations with dominant effects on the behavior and morphology of the nematode Caenorhabditis elegans. Genetics 113**:** 821–852.

Patel, N., D. Thierry-Mieg and J. R. Mancillas, 1993 Cloning by insertional mutagenesis of a cDNA encoding Caenorhabditis elegans kinesin heavy chain. Proceedings of the National Academy of Sciences 90**:** 9181–9185.

Pellet, J. B., J. A. Haefliger, J. K. Staple, C. Widmann, E. Welker *et al.*, 2000 Spatial, temporal and subcellular localization of islet-brain 1 (IB1), a homologue of JIP-1, in mouse brain. Eur J Neurosci 12**:** 621–632.

Sagasti, A., N. Hisamoto, J. Hyodo, M. Tanaka-Hino, K. Matsumoto *et al.*, 2001 The CaMKII UNC-43 activates the MAPKKK NSY-1 to execute a lateral signaling decision required for asymmetric olfactory neuron fates. Cell 105**:** 221–232.

Schindelin, J., I. Arganda-Carreras, E. Frise, V. Kaynig, M. Longair *et al.*, 2012 Fiji: an open-source platform for biological-image analysis. Nature Methods 9**:** 676–682.

Siete, C., R. Xiong, A. Khalid, Y. W. Hsieh and C. F. Chuang, 2024 Immobilization of C. elegans with different concentrations of an anesthetic for time-lapse imaging of dynamic protein trafficking in neurons. MicroPubl Biol 2024.

Thompson, O., M. Edgley, P. Strasbourger, S. Flibotte, B. Ewing *et al.*, 2013 The million mutation project: a new approach to genetics in Caenorhabditis elegans. Genome Res 23**:** 1749–1762.

Troemel, E. R., A. Sagasti and C. I. Bargmann, 1999 Lateral signaling mediated by axon contact and calcium entry regulates asymmetric odorant receptor expression in C. elegans. Cell 99**:** 387–398.

bioRxiv preprint doi: https://doi.org/10.64898/2026.04.30.722091; this version posted May 5, 2026. The copyright holder for this preprint (which was not certified by peer review) is the author/funder, who has granted bioRxiv a license to display the preprint in perpetuity. It is made available under aCC-BY 4.0 International license.

Vanhoven, M. K., S. L. Bauer Huang, S. D. Albin and C. I. Bargmann, 2006 The claudin superfamily protein nsy-4 biases lateral signaling to generate left-right asymmetry in C. elegans olfactory neurons. Neuron 51**:** 291–302.

Xiong, R., J. Yang, S. Yuan, E. Liu, X. Wang *et al.*, 2026 An apical junction protein antagonizes mechanosensitive calcium signaling to establish stochastic choices of olfactory neuron subtypes. bioRxiv.

Supplemental Figure Legends

Figure S1. Expression of TIR-1::GFP in wild type, *jip-1* mutants, and *unc-104* mutants. (**A, B**) Quantification of TIR-1::GFP fluorescence intensity, expressed from a stably integrated transgene *odr-3p::tir-1::GFP*, in the AWC axon (A) and cell body (B) at the L1 stage. AU, arbitrary unit. n, the total number of animals analyzed. Student's *t*-test was used for statistical analysis. Error bars, standard errors of the mean. (**C)** Images of wild type and *unc-104(e1265)* mutants expressing TIR-1::GFP in AWC cells from a stably integrated transgene *odr-3p::tir-1::GFP* in L1. The anterior is left, and the ventral is down. Scale bar, 5 µm. (**D**) Left panels: Images of wild type and *jip-1(vy6)* mutants expressing *odr-3p::GFP* in AWC cells at the L1 stage. The anterior is left, and the ventral is down. Scale bar, 5 µm. Right panel: Quantification of GFP fluorescence intensity in the AWC cell body. In each animal, fluorescence intensity was quantified from the single focal plane with the brightest GFP expression in the AWC cell body. AU, arbitrary unit. n, the total number of animals analyzed. Student's *t*-test was used for statistical analysis. ns, not significant. Error bars, standard errors of the mean.

bioRxiv preprint doi: https://doi.org/10.64898/2026.04.30.722091; this version posted May 5, 2026. The copyright holder for this preprint (which was not certified by peer review) is the author/funder, who has granted bioRxiv a license to display the preprint in perpetuity. It is made available under aCC-BY 4.0 International license.

Figure S2. Gene structure of *jip-1*. (**A**) Top: Genomic DNA structure of *jip-1* isoforms. PID, phosphotyrosine-interaction domain. The arrowhead indicates the position at which the amino acid sequence differs between *C. elegans* JIP-1 protein isoforms a/e/f (SFFSPD) and b/c/d (Y). Bottom: Structure of transgenes used to rescue *jip-1(vy6)* mutants and the analysis of *jip-1* expression pattern. (**B**) Amino acid sequence alignment of PID domain in *Caenorhabditis elegans* (Ce) JIP-1 with homologs in *Drosophila melanogaster* (Dm) (PELLET *et al.* 2000), *Mus musculus* (Mm), and *Homo Sapiens* (Hs). The alignment was performed using UniProt (https://www.uniprot.org/align/). Red, identical; blue, similar; black, not similar.

Figure S3. UNC-104 and UNC-116 are required for the dynamic trafficking of JIP-1 in the AWC axon. (**A**) Representative kymographs of GFP::JIP-1a and GFP::JIP-1a^{E58K} movement in the AWC axon at the L1 stage. (**B, C**) Quantification of the percentage of anterograde and retrograde GFP::JIP-1a and GFP::JIP-1a^{E58K} trafficking events (B) and the average velocity of trafficking events (C). Asterisks indicate significant differences ($p < 0.0001$) in the same direction of movement between GFP::JIP-1a versus GFP::JIP-1a^{E58K} or GFP::JIP-1a in wild type versus *unc-104(e1265)* or *unc-116(e2310)* mutants. *P*-values were determined by a *Z*-test (B) or Student's *t*-test (C). Error bars represent the standard error of the proportion (B) or the standard error of the mean (C). ns, not significant. n, number of trafficking events.

bioRxiv preprint doi: https://doi.org/10.64898/2026.04.30.722091; this version posted May 5, 2026. The copyright holder for this preprint (which was not certified by peer review) is the author/funder, who has granted bioRxiv a license to display the preprint in perpetuity. It is made available under aCC-BY 4.0 International license.

Figure S1

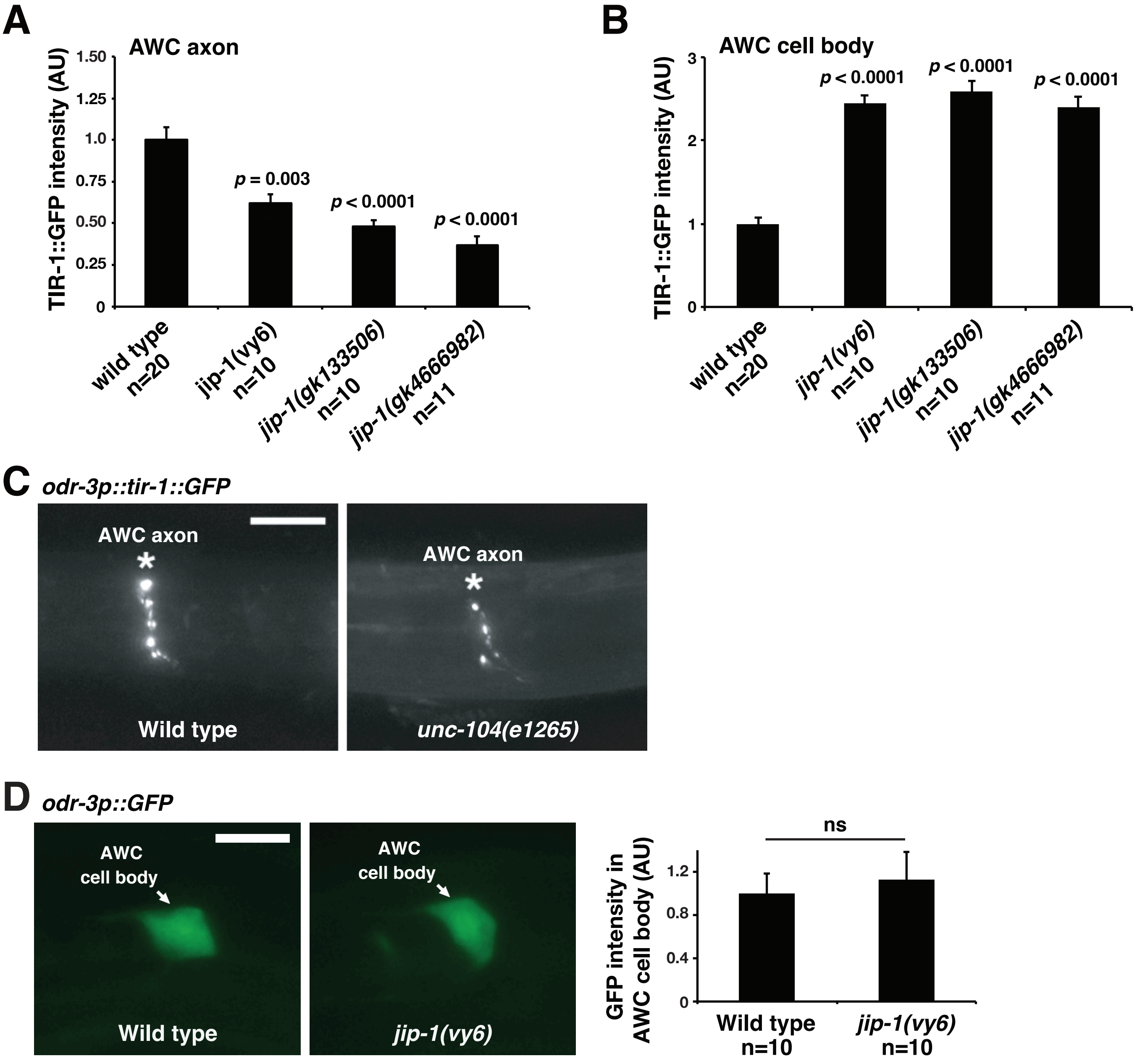

bioRxiv preprint doi: https://doi.org/10.64898/2026.04.30.722091; this version posted May 5, 2026. The copyright holder for this preprint (which was not certified by peer review) is the author/funder, who has granted bioRxiv a license to display the preprint in perpetuity. It is made available under aCC-BY 4.0 International license.

Figure S2

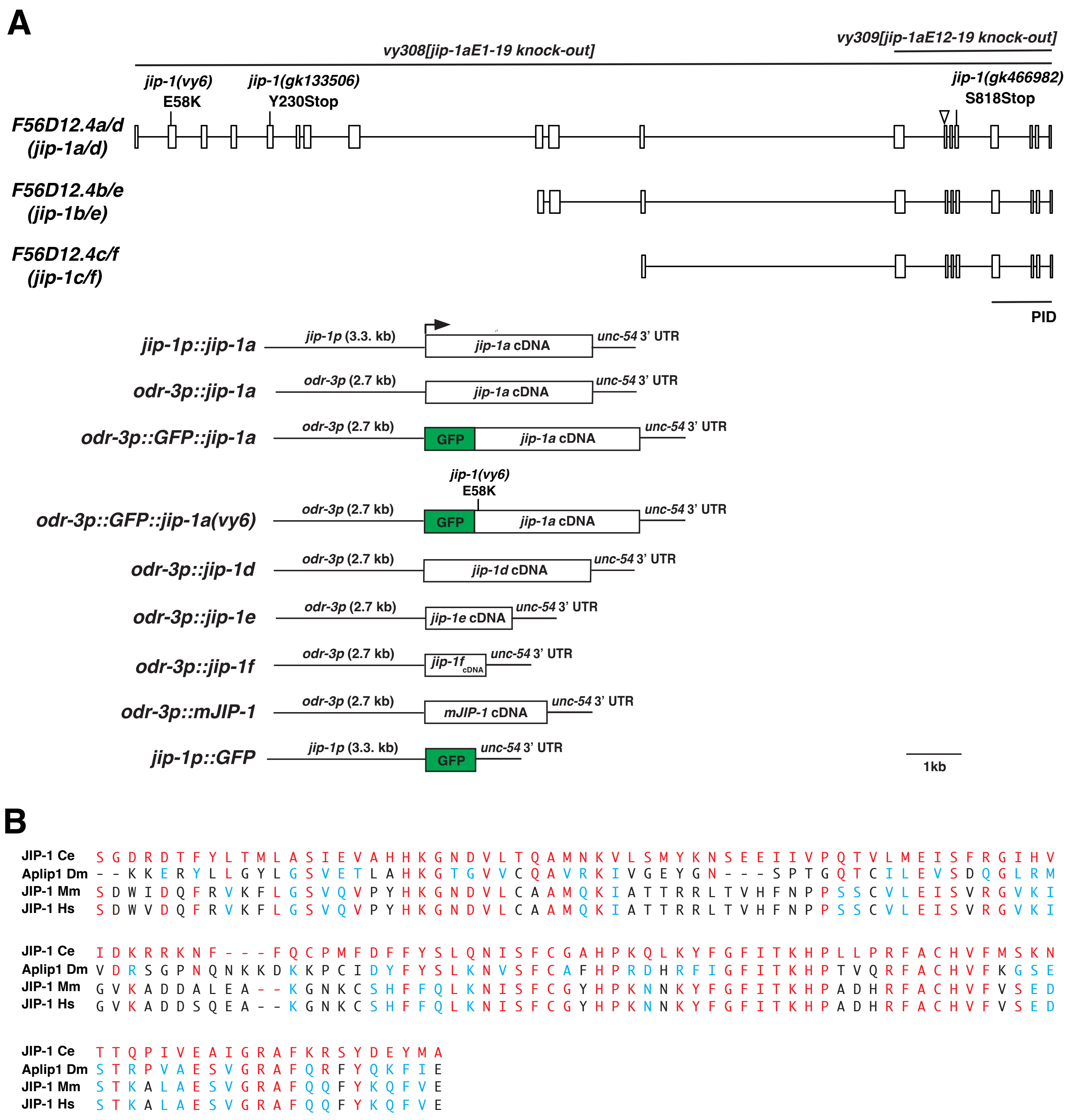

Figure S3

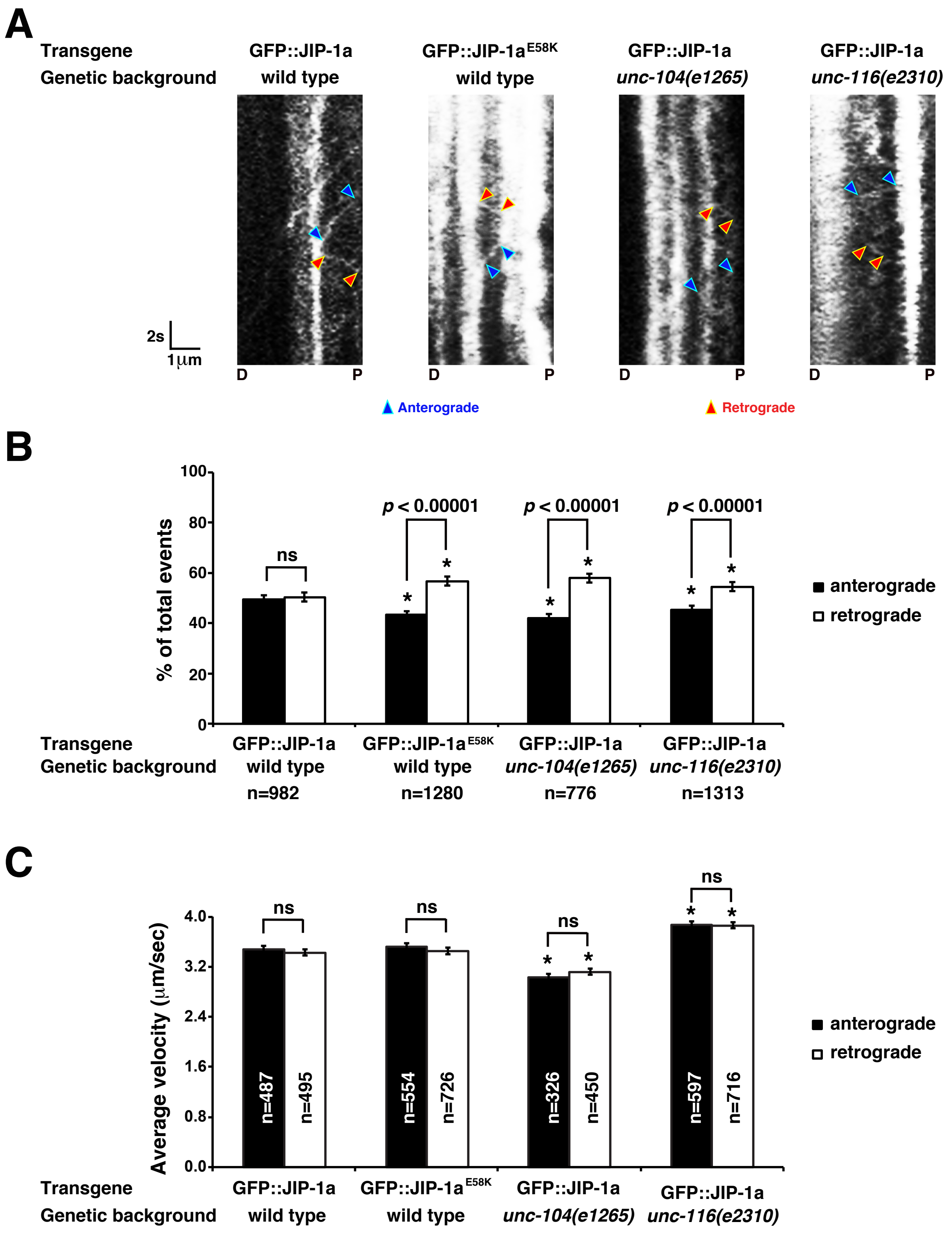

A
Transgene
Genetic background
GFP::JIP-1a
wild type
GFP::JIP-1a E58K
wild type
GFP::JIP-1a
unc-104(e1265)
GFP::JIP-1a
unc-116(e2310)
2s
1µm
D
P
Anterograde
Retrograde
B
% of total events
100
80
60
40
20
0
ns
p < 0.00001
p < 0.00001
p < 0.00001
anterograde
retrograde
Transgene
Genetic background
GFP::JIP-1a
wild type
n=982
GFP::JIP-1a E58K
wild type
n=1280
GFP::JIP-1a
unc-104(e1265)
n=776
GFP::JIP-1a
unc-116(e2310)
n=1313
C
Average velocity (µm/sec)
4.0
3.2
2.4
1.6
0.8
0.0
ns
ns
ns
ns
n=487
n=495
n=554
n=726
n=326
n=450
n=597
n=716
anterograde
retrograde
Transgene
Genetic background
GFP::JIP-1a
wild type
GFP::JIP-1a E58K
wild type
GFP::JIP-1a
unc-104(e1265)
GFP::JIP-1a
unc-116(e2310)
